# 2-APCAs, the Novel Microtubule Targeting Agents Active against Distinct Cancer Cell Lines

**DOI:** 10.3390/molecules26030616

**Published:** 2021-01-25

**Authors:** Sergei Boichuk, Aigul Galembikova, Firuza Bikinieva, Pavel Dunaev, Aida Aukhadieva, Kirill Syuzov, Svetlana Zykova, Nazim Igidov, Alexander Ksenofontov, Pavel Bocharov

**Affiliations:** 1 Department of Pathology, Kazan State Medical University, 420012 Kazan, Russia; ailuk000@mail.ru (A.G.); firuza1995@mail.ru (F.B.); dunaevpavel@mail.ru (P.D.); arom1705@mail.ru (A.A.); grop2019@gmail.com (K.S.); 2 Сentral Research Laboratory, Kazan State Medical University, 420012 Kazan, Russia; 3 Perm State Academy of Pharmacy, 614990 Perm, Russia; zykova.sv@rambler.ru (S.Z.); igidov_nazim@mail.ru (N.I.); 4 G.A. Krestov Institute of Solution Chemistry of the Russian Academy of Sciences, 153045 Ivanovo, Russia; ivalex.09@mail.ru (A.K.); bochpavl@gmail.com (P.B.); 5 Institute of Solution Chemistry, Ivanovo State University of Chemistry and Technology, 153000 Ivanovo, Russia

**Keywords:** microtubules, tubulin, cell cycle, mitotic arrest, apoptosis, soft tissue sarcomas (STS), gastrointestinal stromal tumors (GISTs), breast, lung, and prostate cancer, paclitaxel, pirroles

## Abstract

Microtubules are known as the most attractive molecular targets for anti-cancer drugs. However, the number of serious limitations of the microtubule targeting agents (MTAs) including poor bioavailability, adverse effects (e.g., systemic and neural toxicity), and acquired resistance after initiation of MTA-based therapy remain the driving forces to develop the novel therapeutic agents effectively targeting microtubules and exhibiting potent anti-tumor activities. Here, we report the discovery of 2-amino-pyrrole-carboxamides (2-APCAs), a novel class of MTA, which effectively inhibited the growth of the broad spectrum of cancer cell lines in vitro, including various types of breast, prostate, and non-small lung cancer (NSLC), soft tissue sarcomas (STS) (e.g., leio-, rhabdomyo-, and fibrosarcomas), osteosarcomas and gastrointestinal stromal tumors (GISTs). Importantly, 2-APCAs were also effective in cancer cell lines exhibiting resistance to certain chemotherapeutic agents, including MTAs and topoisomerase II inhibitors. The anti-proliferative effect of 2-APCAs was due to their ability to interfere with the polymerization of tubulin and thereby leading to the accumulation of tumor cells in the M-phase. As an outcome of the mitotic arrest, cancer cells underwent apoptotic cell death which was evidenced by increased expression of cleaved forms of the poly-ADP-ribose polymerase (PARP) and caspase-3 and the increased numbers of Annexin V-positive cells, as well. Among the compounds exhibiting the potent anti-cancer activities against the various cancer cell lines indicated above, 2-APCA-III was found the most active. Importantly, its cytotoxic activities correlated with its highest potency to interfere with the dynamics of tubulin polymerization and inducement of cell cycle arrest in the G2/M phase. Interestingly, the cytotoxic and tubulin polymerization activities of 2-APCAs correlated with the stability of the «tubulin—2-АРСА» complexes, illustrating the “tubulin-2-APCA-III” complex as the most stable. Molecular docking showed that the binding site for 2-АРСА-III is located in α tubulin by forming a hydrogen bond with Leu23. Of note, single-cell electrophoresis (Comet assay) data illustrated the low genotoxic activities of 2-APCAs when compared to certain anti-cancer chemotherapeutic agents. Taken together, our study describes the novel MTAs with potent anti-proliferative and pro-apoptotic activities, thereby illustrating them as a scaffold for the development of successful chemotherapeutic anti-cancer agent targeting microtubules.

## 1. Introduction

The microtubules play an important role in a broad spectrum of cellular processes including proliferation, signaling, trafficking, and migration. For this reason, multiple microtubule-targeting agents (MTAs) have been extensively developed and currently represent one of the oldest and diverse families of chemotherapeutic drugs exhibiting potent anti-cancer activities [1]. Molecular mechanisms of action of MTAs are distinct due to the binding to tubulin and/or microtubules, therefore altering the microtubule polymerization and dynamics in diverse ways. According to the molecular mechanism of action, MTAs are classified into two major types: “microtubule-destabilizing” agents (MDAs) and “microtubule-stabilizing” agents (MSAs). The well-known MDAs (e.g., vinca alkaloids, colchicine, and its analogs) inhibit microtubule polymerization via binding to one of two domains on tubulin, the “vinca” domain, and the “colchicine” domain. Maystansine and pironetin were recently discovered and included in this group due to their ability to inhibit tubulin assembly into microtubules by binding to the sites, which are distinct from the typical vinca alkaloid- and colchicine- binding domains [2,3,4]. The MDAs indicated above exhibit the diverse mechanisms to inhibit a proper microtubule formation, including inserting a weight between the assembled heterodimers, inhibiting the curve-to straight transformation of the tubulin heterodimer, stabilizing the assembly of incompetent polymers or blocking longitudinal interactions [2,5,6]. In contrast to MDAs, MSAs (e.g., taxol, docetaxel) enhance microtubule polymerization and stabilize the microtubules via binding to the taxoid binding site on β-tubulin, which is located on the inside surface of the microtubules [7,8]. Laulimalide [9] and peloruside A [10] are marine sponge products, which were included into MSAs due to their ability to promote tubulin assembly and microtubule stability via binding to the non-taxane site on β-tubulin and use their macrolide structures to interact with the second tubulin dimer across protofilaments [11]. 

Despite the different molecular mechanisms of MTA indicated above, abnormalities in the micro spindle dynamic state induce a permanent G2/M arrest followed by cell death via the activation of divergent apoptotic mechanisms [12]. Given that microtubules are present both in interphase cells and in dividing cells and taking into account that cancer cells exhibit the intensive proliferative rate, cancer cells are expected to be highly sensitive to the therapeutic inhibition of microtubule dynamics. Indeed, the most common classes of MTAs, such as vinca alkaloids and taxanes are currently administrated to patients with a broad spectrum of solid tumors and hematological malignancies. MTAs are most frequently used in combination with chemotherapy regimens, including curative regimens and in the adjuvant settings. Despite these well-known facts, the clinical use of MTAs is limited due to the serious adverse effects including bone marrow toxicity, immunosuppression, and neuropathy [13,14]. Another serious limitation for the extended clinical use of MTAs for cancer therapy is the rapid development of tumor resistance through diverse mechanisms, including the overexpression of ABC-transporters, such as P-glycoprotein [15,16,17], altered expression of specific β-tubulin isotypes [18], mutations in β-tubulin, delay of G2/M transition, impairment of the mitotic checkpoints, and apoptotic pathways which were extensively reviewed [19]. Thus, there is a critical need to develop novel potent and low-toxic anti-cancer agents targeting microtubules and overcoming the resistance to the MTAs currently used for cancer therapy. Of note, the vast majority of MTAs approved for cancer therapy bind to β-tubulin, whereas pironetin is the only compound currently known to bind solely to α-tubulin [3,4], thereby illustrating α-tubulin as a novel attractive molecular therapeutic target for cancers that developed resistance to the classical MTAs which bind to β-tubulin [20].

We showed previously that ethyl-2-amino-pyrrole-3-carboxylates (EAPCs) exhibit the potent anti-cancer activities against a broad spectrum of cancer cell lines both in vitro and in vivo [21,22,23]. The molecular mechanism of action was due to their ability to interfere in the microtubule dynamic state by inhibiting the tubulin polymerization and induction of apoptotic cell death. In this study, we expanded our research of pyrrole-containing heterocyclic compounds. To determine the potential relationship between the structure and biological activities of the heterocyclic compounds, we synthesized the novel compounds. For example, interaction of (Z)-*N*-(5-tert-butyl)-2-oxofuran-3(2*H*)-yliden-2-phenylaminobenzohydrazide (I) with cyanoacetic ester in the presence of triethylamine was used to synthesize the novel derivative (E)-ethyl-2-amino-5-(3,3-dimethyl-4-oxobutyliden)-4-oxo-1-(2-phenylaminobenzamido)-4,5-dihydro-1H-pyrrole-3-carboxylate (II) [24]. We utilized this concept to synthesize chemical compounds exhibiting the potent tubulin-binding properties. As a result, we identified the 2-amino-1-benzamido-4-oxo-4,5-dihydro-1*H*-pyrrole-3-carboxamides (2-APCAs) exhibiting potent cytotoxic and anti-proliferative activities against a broad spectrum of cancer cell lines. The molecular mechanism of action of 2-APCAs was due to the inhibition of tubulin depolymerization, induction of mitotic arrest, and apoptotic cell death. Importantly, 2-APCAs were also active against multi-drug resistant (MDR) cancer cells, thereby illustrating the discovery of a novel potent class of anti-cancer compounds targeting microtubules. 

## 2. Results

### 2.1. 2-APCAs Inhibit the Viability of the Multiple Cancer Cell Lines

**Figure 1:**
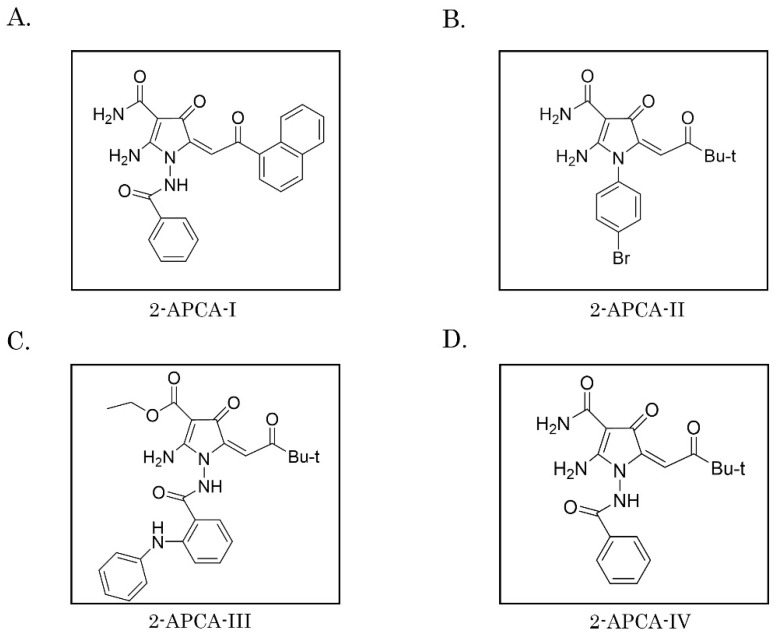
The chemical structures of 2-APCAs are displayed. (**A**) 2-APCA-I; (**B**) 2-APCA-II; (**C**) 2-APCA-III; (**D**) 2-APCA-IV.

To examine whether 2-APCAs synthesized in our laboratory exhibit the growth-inhibitory capacities against cancer cells, we initially performed an MTS-based survival assay with a broad spectrum of cancer cell lines (e.g., HCC1806 and MDA-MB-231 breast cancer, H1299 non-small cell lung cancer (NSCLC), PC-3 prostate cancer, SK-LMS-1 leiomyosarcoma, RD rhabdomyosarcoma, HT-1080 fibrosarcoma, A673 Ewing’s sarcoma, U2-OS and SAOS-2 osteosarcoma, GIST T-1 gastrointestinal stromal tumor). Each cancer cell line was treated with various concentrations of 2-APCAs (0.1–100.0 μM) for 48–72 h. For the positive control, cancer cells were also treated with paclitaxel (PTX) and vinblastine (VIN), the well-known MTAs. Based on the preliminary analysis of 2-APCAs derivatives, we identified four compounds (Figure 1) which were most effective against a broad spectrum of cancer cell lines as indicated above and inhibited their growth in a dose-dependent manner. The IC50 values for 2-APCAs are shown in Table 1 and Table 2.

To further corroborate these findings, we examined whether 2-APCAs are also effective against cancer cell lines that exhibited resistance to certain chemotherapeutic agents, including MTAs, and targeted drug imatinib mesylate (IM) used as a first line drug for GIST therapy. For this purpose, we utilized three multi-drug resistant (MDR) cancer cell lines—HCC1806 Tx-R, GIST T1-Tx-R, or targeted drugs (e.g., imatinib mesylate, IM)—GIST T1-IM-R. All these cell lines were previously generated in our laboratory and characterized by the chemotherapeutic drug(s) uptake/efflux and expression of ABC-transporters (Supplementary Figure S1A–C). Indeed, we observed that 2-APCAs were effective against the cancer cell lines that acquired secondary resistance to the other chemotherapeutic agents, including MTAs. The comparative IC50 values for 2-APCAs in sensitive vs. resistant cancer cell lines are shown in Table 3. The growth inhibitory curves shown in Supplementary Figure S1D,E illustrate that 2-APCAs were equally effective for both PTX-sensitive and resistant HCC breast cancer and GIST T-1 cell lines, respectively. Thus, these data illustrate that 2-APCAs were evenly effective in sensitive and resistant cancer cell lines.

### 2.2. 2-APCAs Induce Mitotic Arrest of Cancer Cells and Enhance Tubulin Polymerization

**Figure 2:**
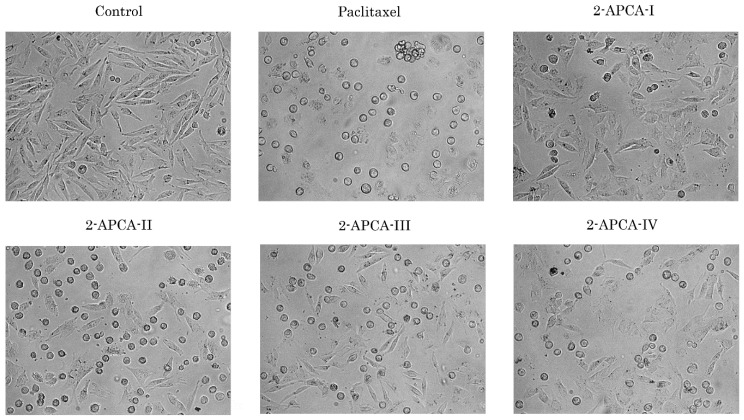
The 2-APCAs induce the changes in morphology of cancer cells. Morphological changes in IM-resistant gastrointestinal stromal tumor (GIST T-1R) cells treated with solvent (DMSO) (negative control), 2-APCAs, and paclitaxel (positive control). Cells were treated with 10 μM of 2-APCAs (I-IV), or 0.1 μM paclitaxel for 24 h and further subjected to the light microscopy (10× magnification).

Given that the majority of 2-APCAs-treated cancer cells acquired a round shape morphology, which was similar to the cells treated with paclitaxel (PTX) (Figure 2), we proposed that growth-inhibitory properties of 2-APCAs are due to their ability to induce mitotic arrest. 

**Figure 3:**
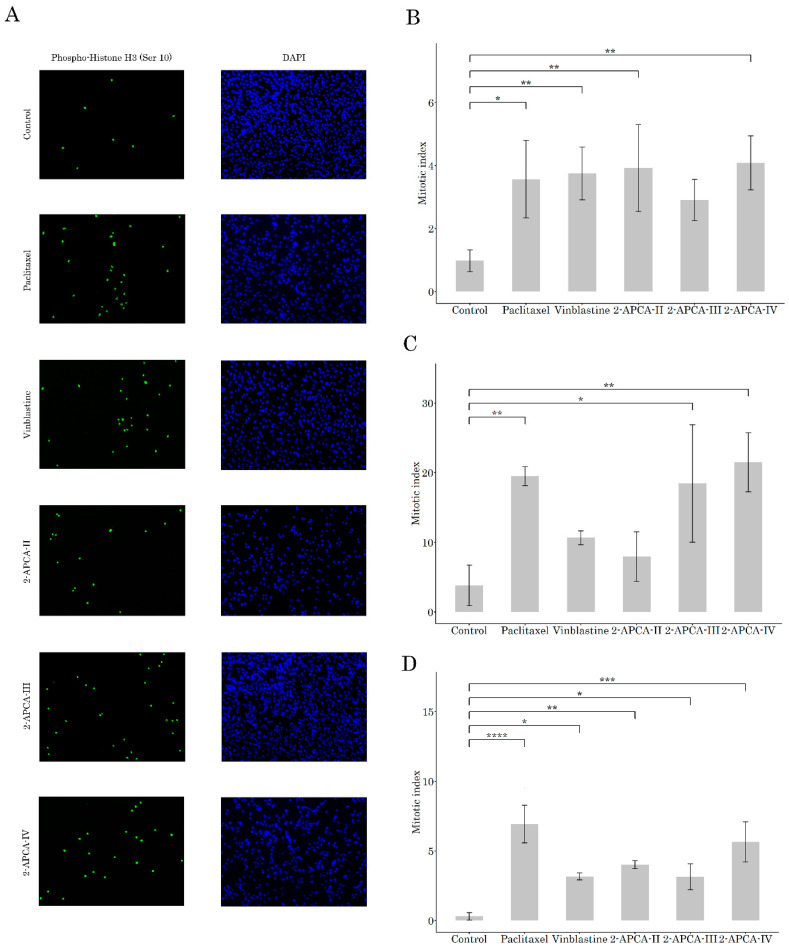
The 2-APCAs induce the accumulation of cancer cells in the M-phase. (**A**) The representative immunofluorescence images (40×) of RD cells treated with DMSO (negative control), 2-APCAs (10 μmol), paclitaxel (0.1 μmol), vinblastine (0.01 μmol), and immunostained using the Alexa488 labeled phospho-histone 3 (Ser10) antibody. DAPI nuclear staining was used to outline an equal number of cells. (**B**) Graph depicting the number of mitotic cells after 2-APCAs treatment from 3 independent experiments. Cells treated with DMSO, paclitaxel, and vinblastine were used as the negative and positive controls, respectively. (**C**) Quantitative analysis of mitotic cells in H1299 lung cancer cells treated with DMSO (control), 2-APCAs, paclitaxel, and vinblastine. (**D**) Quantitative analysis of mitotic cells in MDA-MB-231 breast cancer cells treated as indicated above. * *p* < 0.05; ** *p* < 0.01; *** *p* < 0.001; **** *p* < 0.0001.

To test this hypothesis, we initially performed immunofluorescence staining to examine the expression of Histone 3 phosphorylated at Ser10 residues (pH3Ser10), a well-known marker of mitotic cells. Indeed, we observed a substantial increase in the numbers of pH3Ser10-positive cells in 2-APCA-treated RD rhabdomyosarcoma cells, when compared to non-treated cells (Figure 3A,В). As expected, PTX and VIN also induced a robust increase of mitotic cells in RD cells. Similar results were obtained for the H1299 lung cancer (Figure 3C) and MDA-MB-231 breast cancer (Figure 3D). The accumulation of cancer cells in the M-phase after the 2-APCA treatment was also evidenced by the changes in expression of the cell-cycle regulatory proteins measured by Western blotting. Similar to immunofluorescence staining, we observed a substantial increase of pH3 Ser10 expression in cancer cell lines treated with 2-APCA-III (Supplementary Figure S2). Of note, for MDA-MB-231 breast cancer cell line this effect was more potent when compared with PTX, a common chemotherapeutic agent used for therapies of patients with breast cancer. Conversely, the expression of Mdm2, phospho-Cdk2 Tyr15, and cyclin A2 significantly decreased in 2-APCA-III-treated cells, which was in consistency with the cell cycle abnormalities induced by this compound (Supplementary Figure S2). A high potency of 2-APCA-III to induce the mitotic arrest of cancer cells was also confirmed by the FACS-based cell-cycle analysis, illustrating the increased numbers of cells in the G2/M phase after treatment with 2-APCAs, when compared to the non-treated cells. Again, PTX used as a positive control induced similar abnormalities in the cell cycle distribution in cancer cells. 

**Figure 4:**
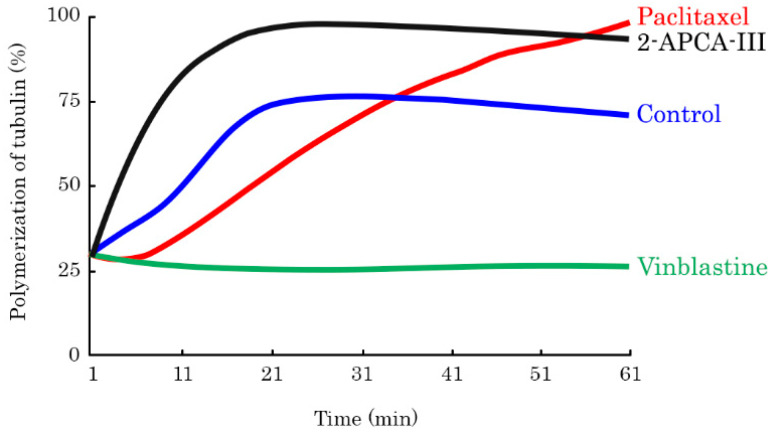
Dynamics of tubulin polymerization in samples treated with 2-APCA-III. Tubulin was also incubated with paclitaxel and vinblastine at 37 °C and absorbance was assessed every minute for 1 h. A shift of the curve to the upper left of the control (DMSO) represents an increase of the polymerized microtubule. A shift to the down right reflects the decrease in the rate of tubulin polymerization.

Given that mitotic arrest might be due to the abnormalities of the microtubule dynamic state, we performed a tubulin polymerization assay to assess the microtubule spindle formation, where an increase in the absorbance at 340 nm indicated an increase in tubulin polymerization. As expected, we observed a significant increase in microtubule polymerization in PTX-treated samples, whereas VIN strongly inhibited tubulin polymerization (Figure 4). We observed the enhanced tubulin polymerization in all four 2-APCAs-treated samples. Moreover, these compounds triggered tubulin polymerization in much earlier time-points when compared to PTX-treated samples. Of note, 2-APCA-III induced a significant increase in tubulin polymerization and was found to be much more effective when compared to PTX (Figure 4). Thus, our data illustrate that 2-APCAs effectively interferes with the microtubules dynamic state.

### 2.3. The 2-APCAs Induce Apoptosis of Breast, Lung, and Prostate Cancer Cells

**Figure 5:**
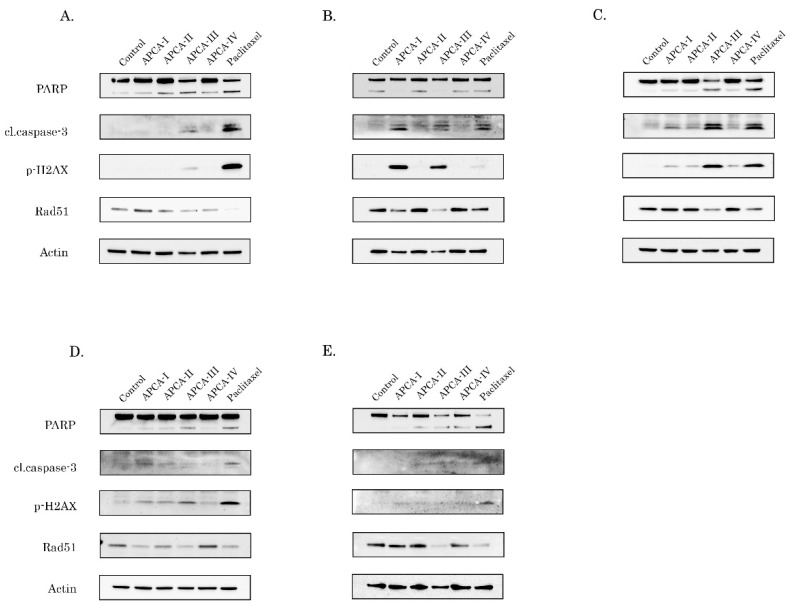
The 2-APCAs induce apoptosis of epithelial cancer cell lines. Immunoblot analysis for apoptosis markers (cleaved forms of PARP and caspase-3), Rad51 recombinase, and γ-H2AX in НСС1806 (**А**), MDA-MB-231 (**B**), H1299 (**C**), PC-3 (**D**), and НеLa (**E**) cells after treatment with DMSO (negative control), 2-APCAs (I-IV), and paclitaxel for 72 h. Actin stain used as a loading control.

To determine whether the decreased viability of 2-APCAs-treated cancer cells was due to the activation of apoptosis as an outcome of mitotic arrest, we initially examined the expression of apoptotic markers (cleaved forms of caspase-3 and PARP). Given that taxanes are chemotherapeutic drugs which are commonly used to treat malignancies with the epithelial origin, we initially examined the pro-apoptotic effect of 2-APCAs on breast cancer cells. Taking into account that the chemotherapeutic agents are the only therapeutic option for patients with triple-negative breast cancer due to the lack of specific molecular targets (e.g., expression of HER2-neu, or estrogen/progesterone receptors), we focused primarily on the triple-negative breast cancer (TNBC) cell lines (e.g., HCC1806 and MDA-MB-231). We observed a substantial increase of apoptotic markers in both breast cancer cell lines after the 2-APCAs treatment, and (in agreement with our polymerization assay data) 2-APCA-III was found to be most effective against both TNBC cells (Figure 5A,B). This was in concordance with the tubulin polymerization assay data shown in Figure 4. As expected, HCC1806 and MDA-MB-231 cancer cells also underwent apoptotic cell death after the PTX treatment. Similar to the breast cancer cell lines, 2-APCAs were also effective against the other epithelial cancer cell lines. For example, an increased expression of apoptotic markers was observed in 2-APCAs-treated H1299 non-small cell lung cancer, PC-3 prostate cancer, and HeLa cervical cancer cell lines and the pro-apoptotic effects of most 2-APCAs were similar to PTX-treated cells (Figure 5C–E, respectively).

### 2.4. The 2-APCAs Are Effective against Osteosarcomas (OS), STS, and GIST

**Figure 6:**
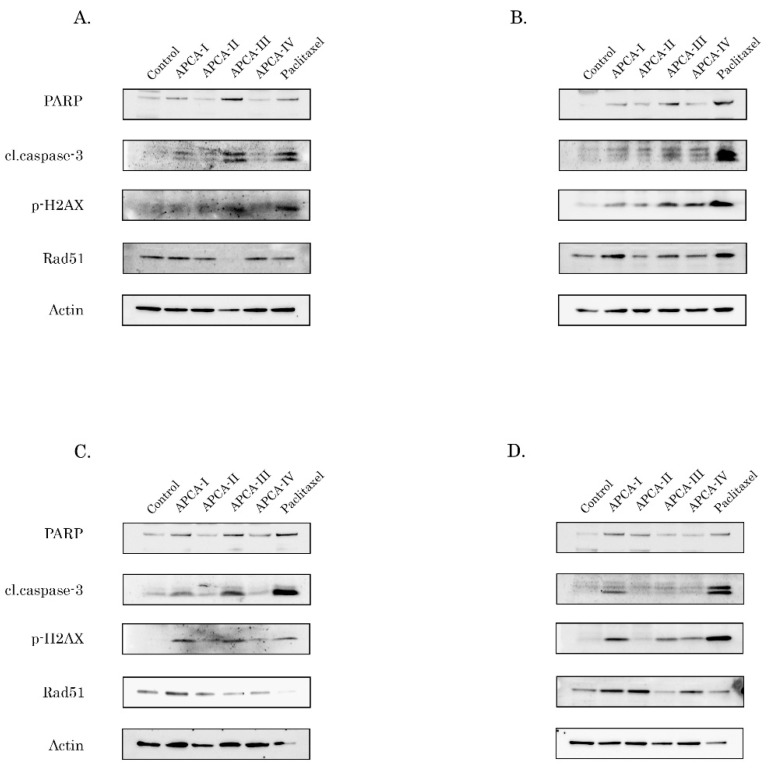
The 2-APCAs induce apoptosis of osteosarcoma (OS) and soft tissue sarcoma (STS) cell lines. Immunoblot analysis for apoptosis markers (cleaved forms of PARP and caspase-3), Rad51 recombinase, and γ-H2AX in U2-OS (**А**), SAOS-2 (**B**), A673 Ewing’s sarcoma (**C**), HT-1080 fibrosarcoma (**D**) cells after treatment with DMSO (negative control), 2-APCAs (I-IV), and paclitaxel for 72 h. Actin stain used as a loading control.

We also found a strong pro-apoptotic activity of 2-APCAs against the other cancer cell lines, exhibiting another tissue origin—soft tissue sarcomas (STS), osteosarcomas (OS), and GIST. Indeed, a substantial increase of cleaved PARP and caspase-3 expression was observed for 2-APCAs-treated OS cell lines (U2-OS and SAOS-2) (Figure 6A,B, respectively), and STS cell lines: For example, A-673 Ewing’s sarcoma and HT-1080 (Figure 6C,D, respectively). Of note, the pro-apoptotic effect of 2-APCAs was dose-dependent, as shown for RD rhabdomyosarcoma, SK-LMS-1 leiomyosarcoma, and GIST T-1R cells (Supplementary Figure S3A,C,E, respectively). The densitometric analysis revealed the significant and dose-dependent changes in expression of apoptotic markers after the 2-APCAs treatment in all types of cancer cell lines, as indicated above (Supplementary Figure S3B,D,F).

**Figure 7:**
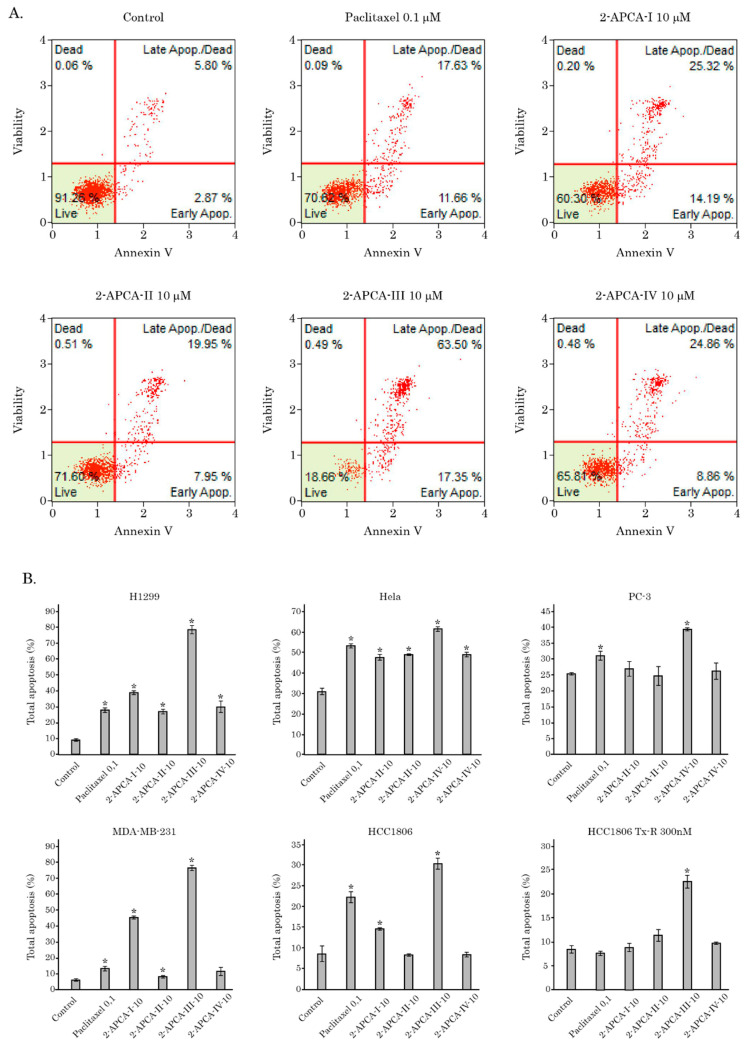
FACS analysis for apoptotic markers in cancer cells treated with 2-APCAs or PTX. (**A**) Representative dot-plots histograms illustrating an increase of early (annexin V-positive/propidium iodide (PI)-negative) and late (double-positive) apoptotic cells in the HCC1806 TNBC cancer cell line treated for 24 h with DMSO (control), 2-APCAs (I-IV), and paclitaxel (positive control). (**B**) Quantitative analysis of apoptotic cells after treatment with DMSO (control), 2-APCAs (I-IV), and PTX (positive control) for 24 h. * *p* < 0.05.

**Figure 8:**
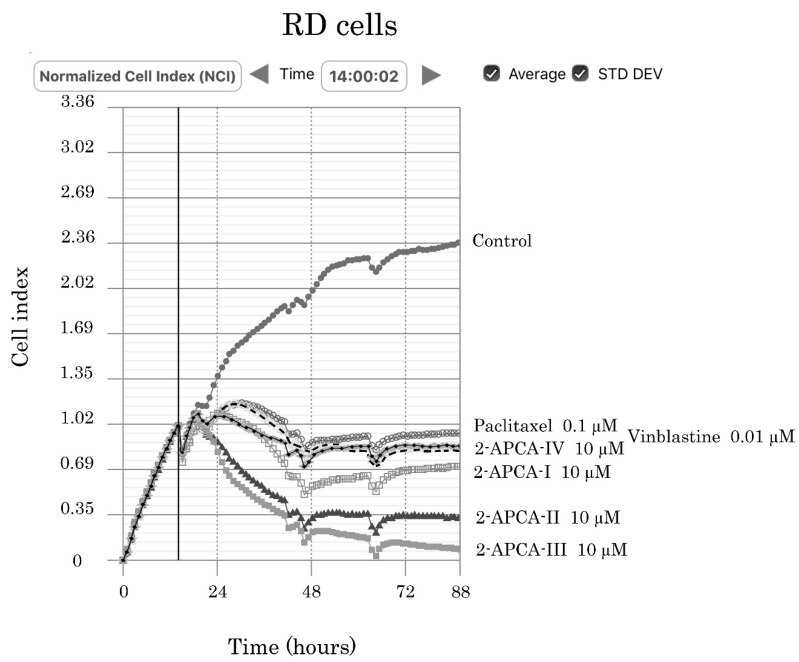
Changes in growth kinetics of RD rhabdomyosarcoma cells treated with DMSO (control) and 2-APCAs (I-IV). Paclitaxel and vinblastine were used as a positive control.

The pro-apoptotic activity of 2-APCAs was also confirmed by the FACS analysis that illustrated a significant increase of Annexin V-positive (i.e., early apoptotic) cells after the 2-APCAs treatment (Figure 7). The total amount of apoptotic cells (early and late apoptotic) was also significantly increased after the 2-APCAs treatment. A similar effect was observed for a broad spectrum of cancer cell lines with different tissue origins, as shown in Figure 7B. Lastly, we found that 2-APCAs significantly inhibited the growth kinetics in cancer cell lines, in particular RD rhabdomyosarcoma cells, as shown in Figure 8.

Collectively, these data illustrate that the decreased viability of 2-APCAs-treated cancer cells is due to their ability to induce mitotic arrest as a result of enhanced tubulin polymerization. This, in turn, activates the apoptotic program, reduces the proliferation speed, and induces cell death of the multiple cancer cell lines, including cell lines, exhibiting resistance to the certain chemotherapeutic agents, including MTAs.

### 2.5. 2-APCAs Induce an Increased Expression of γ-H2AX without DNA Damage

Finally, we examined whether the pro-apoptotic activity of 2-APCAs in cancer cells indicated above was due to their ability to induce DNA damage. Indeed, we observed a substantial increase in the expression of H2AX phosphorylated at residue 139 (γ-H2AX) after the 2-APCAs treatment (Figure 5 and Figure 6 and Supplementary Figure S3), which is known as a common marker of DNA double-strand breaks (DSBs) [25]. Thus, these data suggested the potential DNA-damaging activities for 2-APCAs. Of note, a significant decrease of Rad51 recombinase expression was also observed for the majority of cancer cells treated with 2-APCA-III (Figure 4 and Figure 5). In turn, this correlated with an increase of the apoptotic markers indicated above, thereby suggesting that the potent pro-apoptotic activity of this particular compound might be due to the decreased efficiency of homology-mediated DNA repair mechanisms. Further studies are needed to elucidate the mechanisms of Rad51 decrease in 2-APCAs-treated cells. 

**Figure 9:**
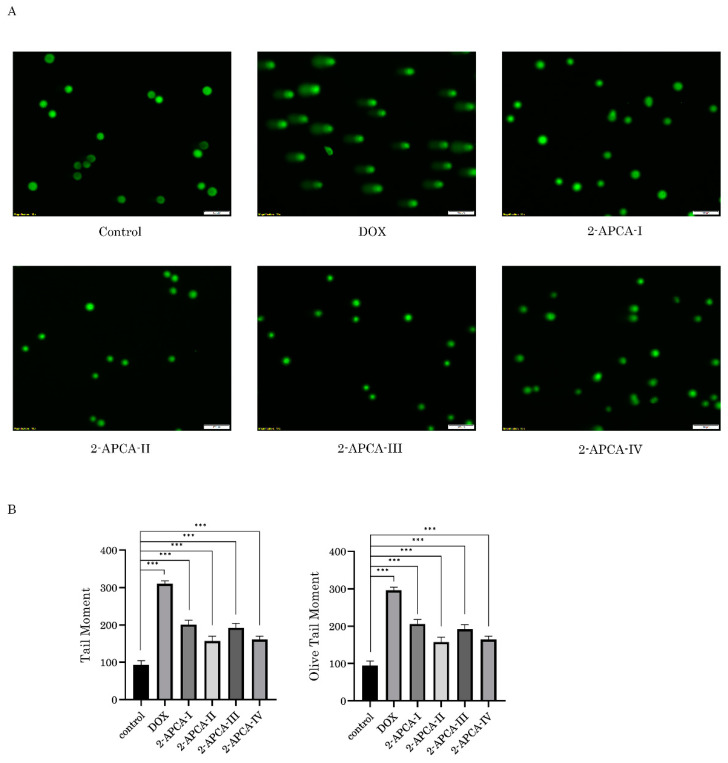
Lack of DNA-damaging activity of 2-APCA. (**A**) Representative images of comets from cells treated with DMSO (control), 2-APCAs (I-IV), and doxorubicin (DOX) (positive control) (scale bars = 100 μm). (**B**) Graphic depiction of the calculated tail moment (TM) and olive tail moment (OTM) from the alkaline comet assay shown in Figure 9A. Columns, mean of at least three independent experiments with a minimum of 50 cells counted per each experiment; bars, SE. *** *p* < 0.001.

It is also known that in addition to the DNA damage, an increased γ-H2AX expression might be due to the alternative mechanisms, in particular, due to the cell cycle abnormalities and accumulation of cells in the M-phase [26]. To delineate between these possibilities, we performed the alkaline-based version of single-cell electrophoresis to determine whether 2-APCAs-treated MDA-MB-231 cells exhibit DNA DSBs. Doxorubicin (DOX) was used as a positive control for these experimental conditions. As shown in Figure 9, minor changes in tail moment (TM) and olive tail moment (OTM) were found between control (non-treated) and 2-APCAs-treated cells, thereby illustrating minimal genotoxic activities of these compounds. As expected, DOX-induced the substantial increase of both parameters indicated above. Similar results were obtained for HCC1806 breast cancer and RD rhabdomyosarcoma cell lines. 

### 2.6. Molecular Docking 

**Figure 10:**
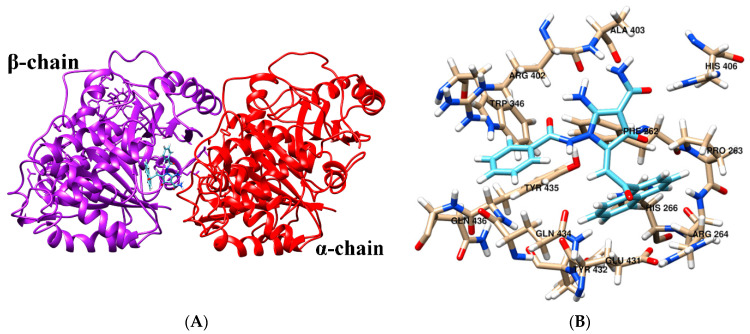

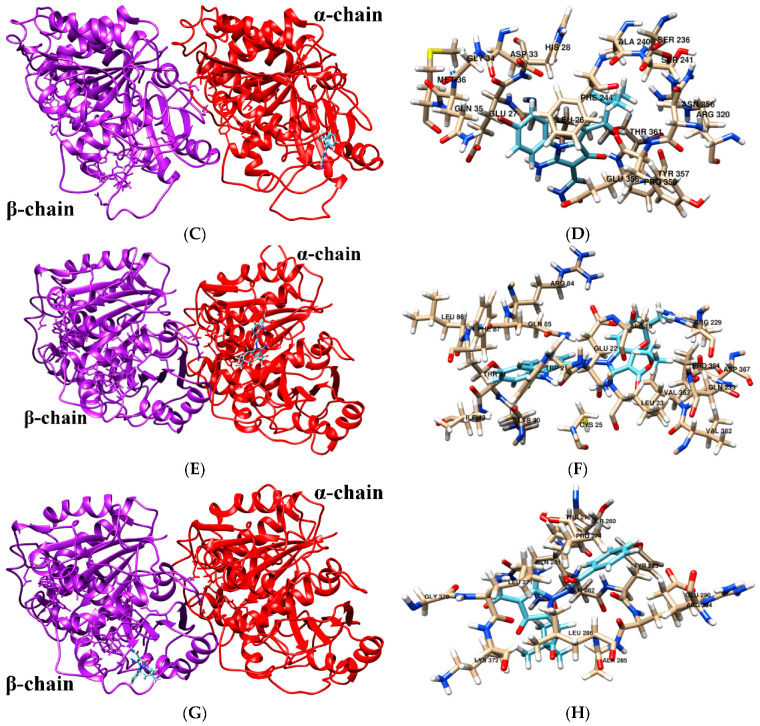
The molecular docking results and binding sites of 2-АРСА-I (**A**,**B**), 2-АРСА-II (**C**,**D**), 2-АРСА-III (**E**,**F**), and 2-АРСА-IV (**G**,**H**) in tubulin.

**Figure 11:**
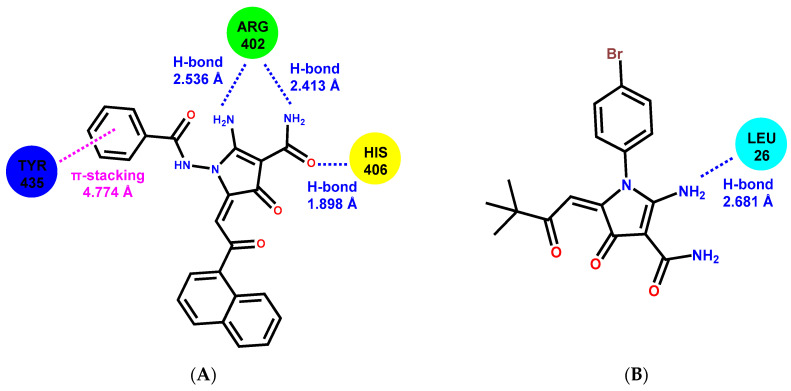

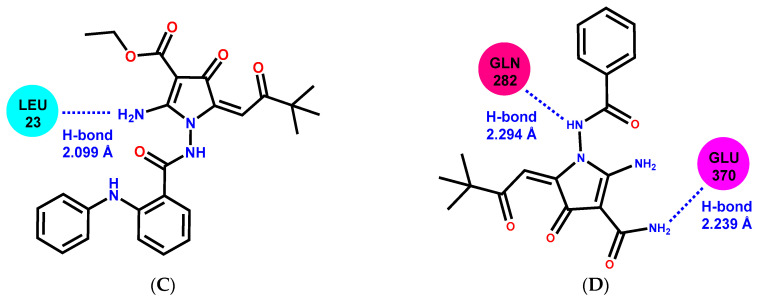
Schematic representation of the interaction of 2-APCA-I (**A**), 2-APCA-II (**B**), 2-APCA-III (**C**), and 2-APCA-IV (**D**) with amino acid residues of binding sites in tubulin.

The molecular docking was performed to find potential binding sites for 2-АРСАs in tubulin. The molecular docking results showed that 2-АРСА-I prefers binding in a pocket at the interface of α- and β-tubulin (Figure 10A,B). The 2-АРСА-II (Figure 10C,D) and 2-АРСА-III (Figure 10E,F) bind in a pocket located in α-tubulin, and 2-АРСА-IV binds in a pocket located in β-tubulin (Figure 10G,H). The amino acid composition of the 2-APCAs binding sites in tubulin is shown in Table 4. We found that 2-APCA-I forms the hydrogen bonds with residues α/ARG402 and α/HIS406, and also participates in π-π-interaction with residue β/TYR435 (Figure 11A). The 2-АРСА-II (Figure 11B) and 2-АРСА-III (Figure 11C) are retained in the binding site of α-tubulin by forming a hydrogen bond with LEU26 and LEU23, respectively. The hydrogen bond with GLN282 and GLU370 is formed by 2-АРСА-IV (Figure 11D). The analysis of free binding energies indicated that the stability of the «tubulin—2-АРСА» supramolecular systems decreased in the series 2-АРСА-III—2-АРСА-II—2-АРСА-I—2-АРСА-IV (Table 5). This was consistent with the highest tubulin polymerization and pro-apoptotic activities observed for 2-APCA-III, as shown in Figure 4, Figure 5 and Figure 6. 

## 3. Discussion

Despite the first members of microtubule-binding agents, including vinca alkaloids and taxanes which were identified almost 50 years ago [27,28], these drugs are currently used for the anti-cancer therapy of broad-spectrum malignancies including solid tumors and hematological malignancies. For example, paclitaxel (PTX) PTX was approved by the US Food and Drug Administration (FDA) as Taxol (^®^) for therapy of advanced ovarian cancer in 1992 and 2 years later for the treatment of metastatic breast cancer. Taxanes currently represent widely active chemotherapeutic agents in many human cancer types including breast, ovarian, and non-small cell lung cancers, malignant brain tumors, and a variety of other solid tumors. Indeed, PTX was found to be very effective in combination during adjuvant therapy for breast cancer. Phase 3 trials showed a clear survival advantage for patients receiving adjuvant paclitaxel-anthracycline combination therapies when compared to similar patients treated with anthracyclines alone [29,30]. Importantly, the PTX benefit was greatest for patients with more aggressive, triple-negative tumors [31]. The effectiveness of the taxanes used as single agents or in combination with a platinum compound in the treatment of advanced NSCLC has been established [32,33]. Taxanes were also shown to have a profound impact on the management of genitourinary tumors, including bladder, prostate, and testis cancers [34,35,36]. For example, in a metastatic setting, PTX used in combination chemotherapy regimens significantly improved the survival of patients with bladder cancer, whereas in metastatic prostate cancer, docetaxel was also shown to improve survival after the development of castration resistance. Vinca alkaloids (e.g., vinblastine, vincristine) are currently used for the treatment of Hodgkin and non-Hodgkin lymphomas, germ cell tumors, testicular carcinoma, breast cancer, etc. The effectiveness of MTA for therapy of STS is less impressive and remains controversial, therefore doxorubicin and ifosfamide-based therapies continue to be the most widely used regimens in the treatment of STS [37,38]. Nevertheless, these therapeutic regimens are noncurative and have a limited benefit when compared with single-agent doxorubicin [39]. Therefore, the efficiency of several additional treatment regimens was extensively assessed over the last decade. Although taxanes were shown to be low-effective in most subtypes of STS as a single-agent [40,41,42,43], the combination regimens of gemcitabine and docetaxel (GD) has shown activity in specific subsets of STS. For example, phase 2 studies of GD-based therapy showed 53% of overall response rates for patients with uterine leiomyosarcomas [44]. Nevertheless, data illustrating the benefits of GD-based therapy for patients with STS are conflicting. For example, a trial conducted by the Sarcoma Alliance for Research through Collaboration (SARC) illustrated a 2-fold increase of response rate (8% vs. 16%) for uterine leiomyosarcomas (LMS) treated with gemcitabine alone or in combination with docetaxel, respectively. The overall survival was also improved for patients receiving GD-based therapy when compared with the gemcitabine arm (12.9 vs. 11.5 months, respectively) [45]. However, the results of the other trial, conducted by the French Sarcoma Group did not confirm the benefits of combination therapy for patients with uterine and non-uterine LMS who had previously received doxorubicin treatment [46]. Similarly, a comparative phase 3 study (GEDDIS trial) revealed low benefits for patients with STS receiving the combination therapy of gemcitabine and docetaxel or single-agent doxorubicin. The progression-free survival rates (PFS) were ~46% at 24 weeks for both STS groups, but the toxicity of the GD therapy was worse when compared with doxorubicin as a single agent [30]. The study conducted on the small number of patients with STS (n = 9) illustrated clear benefits for patients with STS in terms of PFS, clinical benefit rate (CBR), etc. when compared to the single-agent pazopanib activity shown in the PALETTE trial [47]. Importantly, the toxicity profile was also acceptable for this combination of therapy.

Collectively, MTAs might be effective as a single agent but most frequently used in combination chemotherapy regimens, including curative regimens and in the adjuvant settings. However, MTAs-based therapies are usually associated with a number of serious limitations, including poor bioavailability and serious adverse effects (e.g., systemic and neural toxicity, myeloid toxicity, neutropenia, etc.). Moreover, the rapid development of tumor resistance to MTAs via diverse molecular mechanisms also limits their clinical effectiveness. This includes mutations of TUB, selection of tubulin isotypes and its post-translational modifications [18,48], an increased efflux of MTAs due to the upregulation of the MDR1 gene [49], etc. Thus, a broad toxicity profile of MTAs and the rapidly developing resistance remain the main driving forces to develop the novel therapeutic agents effectively targeting microtubules and exhibiting potent anti-tumor activities.

Our present data illustrate potent anti-cancer activities of 2-APCAs against a broad spectrum of cancer cell lines, including breast, lung, and prostate cancer, soft tissue sarcomas, osteosarcoma, and gastrointestinal stromal tumors, as well (Table 1 and Table 2). Importantly, anti-proliferative, and cytotoxic activities of 2-APCAs were also found for cancer cell lines, exhibiting resistance to the certain chemotherapeutic drugs, including paclitaxel (PTX), and targeted drugs, such as IM (Table 3). Next, we found that the anti-cancer activity of 2-APCAs was due to their ability to induce a robust cell cycle arrest in the G2/M phase and the accumulation of cells in the M-phase, which was evidenced by a substantial accumulation of pH3Ser10-positive cells after the 2-APCAs treatment (Figure 3A–E). As expected, this was also associated with the decreased expression of Mdm2, phospho-Cdk-Tyr15, and cyclin A2 (Supplementary Figure S2). The selective accumulation of cancer cells in the M-phase after the 2-APCA treatment was due to their ability to interfere with the microtubules dynamic state and accelerate the tubulin polymerization. 2-APCA-III was found to be the most potent compound that polymerized tubulin in vitro (Figure 4). This was consistent with the analysis of free binding energies which illustrated the highest stability of the «tubulin—2-АРСА-III» supramolecular system. Free binding energies were decreased in the series 2-АРСА-III—2-АРСА-II—2-АРСА-I—2-АРСА-IV, as shown in Table 5. Molecular docking data illustrated the binding sites in tubulin for 2-APCAs. Interestingly, the potential binding sites for 2-APCA-II and -III were found in β-tubulin (Figure 10). Given that the MTAs which are currently approved by FDA for anti-cancer therapy bind exclusively to β-tubulin, and taking into account the broad spectrum of the mechanisms of acquired resistance to MTAs, the development of the anti-mitotic drugs exhibiting the novel molecular target (i.e., β-tubulin) looks attractive and suggests 2-APCAs as a novel and perspective class of MTAs.

As an outcome of the mitotic arrest, 2-APCAs-treated cells underwent apoptotic cell death, which was evidenced by a substantial increase of cleaved forms of caspase-3 and PARP in the vast majority of cancer cell lines (Figure 4 and Figure 5, Supplementary Figure S2). This was also associated with a substantial increase in γ-H2AX expression in most of the cancer cell lines treated with 2-APCAs (Figure 4 and Figure 5, Supplementary Figure S2). Given that γ-H2AX is known as a common surrogate marker of DNA DSBs [9], or might be due to the other cellular physiological processes lacking DNA damage and might be related to the cell cycle phases [26], we performed single-cell electrophoresis to delineate these possibilities. Of note, single-cell electrophoresis data illustrated low genotoxic activities of 2-APCAs (Figure 6), which might be considered as a prospective therapeutic benefit for these compounds exhibiting a lower toxicity profile when compared to the certain chemotherapeutic DNA-damaging agents. Given that 2-APCAs-treated cells exhibited significant cell cycle abnormalities (i.e., accumulation of cancer cells in the M-phase), the increased expression of γ-H2AX might be a consequence of the selective accumulation of cancer cells in the M-phase, as was evidenced earlier [26]. Another interesting fact observed for 2-APCAs-treated cancer cells was the significant decrease of Rad51 recombinase. This was typical for the vast majority of cancer cells treated with 2-APCA-III, exhibiting the potent tubulin-polymerizing and pro-apoptotic properties. Of note, PTX also reduced Rad51 expression in the vast majority of cancer cell lines utilized for the present study (Figure 5 and Figure 6), thereby suggesting the potential link between the microtubules dynamic state and Rad51 expression. Despite the fact that the precise mechanisms of this fact remain to be elucidated, recent findings indicate the potential relationship between tubulin and Rad51. Indeed, MTA induced the retention of DNA damage repair proteins in the cytoplasm, thereby attenuating an effective repair of DNA damage induced by certain chemotherapeutic drugs and providing the molecular mechanism of the rationale use of combined therapies for cancer patients [50]. Further studies revealed an ability of tubulin-binding agents to impair homology-mediated, but not non-homology end-joining (NHEJ)-mediated DNA repair by reduction in the number of cells with Rad51-positive γ-H2AX foci after an external DNA damage [51]. Importantly, the physical association of Rad51 with microtubules was revealed in a co-immunoprecipitation assay, thereby indicating that an efficient response to DNA damage involves a cytoplasmic-to-nuclear transport of Rad51 and might be tubulin-dependent [51]. Lastly, Rad51 recombinase was proposed to be a key factor protecting mitotic chromatin by promoting the mitotic DNA synthesis of the underreplicated DNA [52], thereby suggesting that cancer cells lacking this DDR protein might be extremely sensitive to mitotic DNA damage when the cells enter mitosis before completion of DNA replication [53]. Thus, the ability of 2-APCAs to induce apoptosis of cancer cells might be due to the dual mode of action—mitotic arrest due to the enhancement of tubulin polymerization, which in turn substantially reduces Rad51 expression and triggers the cancer cell death due to the unrepairable mitotic DNA damage. Further studies are required to elucidate this proposal.

Collectively, our present data illustrate that 2-APCAs exhibit the potent anti-cancer activities against a broad spectrum of cancer cell lines in vitro, due to their ability to interfere with the microtubule dynamic state, accumulation of cancer cells in the M-phase, and induction of apoptotic cell death as a consequence of mitotic arrest. In turn, this indicates that 2-APCAs might be considered as a scaffold for the development of a novel and perspective class of MTAs. 

## 4. Materials and Methods

### 4.1. Chemical Compounds

2-APCAs were synthesized in our laboratories according to the standard protocols, as shown elsewhere [24]. Doxorubicin (Dox) were purchased from SelleckChem (Houston, TX, USA), paclitaxel (PTX), and vinblastine (Vin) were obtained from Sigma-Aldrich (St Louis, MO, USA). All the chemicals indicated above were dissolved in 100% dimethyl sulfoxide (DMSO). 

### 4.2. Cell Lines and Culture Conditions

The following human tumor cell lines were used in the present study: HСС1806 parental vs. PTX-resistant breast cancer cell lines, MCF-7 and MDA-MB-231 breast cancer cells, PC-3 prostate cancer cell line, H1299 non-small lung cancer (NSLC) cell line, SK-LMS-1 leiomyosarcoma, RD rhabdomyosarcoma, HT1080 fibrosarcoma, A-673 Ewing’s sarcoma, SAOS-2 and U-2 OS osteosarcoma cell lines, gastrointestinal stromal tumor parental GIST-T1 vs. PTX- and imatinib mesylate (IM)-resistant cell lines, normal human diploid fibroblast cells (BJ). HСС1806, MCF-7, MDA-MB-231, PC-3, H1299, SK-LMS-1, RD, HT-1080, A-673, SAOS-2, and U-2 OS tumor cell lines were obtained from the American Type Culture Collection (Manassas, VA, USA). The human GIST-T1 cell line was a generous gift from Dr T. Taguchi (University of Kochi, Kochi, Japan) [54]. BJ tert human fibroblasts were kindly provided by O. Gjoerup, University of Pittsburgh, USA. The IM-resistant GIST T-1R subline was established in our laboratory after a continuous induction from 0.4 to 1000 nM IM in a stepwise increasing concentration manner [55]. PTX-resistant HCC1806-Tх-R and GIST T-1-Тx-R sublines were established in our laboratory after a continuous induction from 0.001 to 300 nM PTX in a stepwise increasing concentration manner [56,57]. All cell lines were maintained in Dulbecco’s modified Eagle’s medium or RPMI-1640 medium, supplemented with 10–15% fetal bovine serum, 1% L-glutamine, 50 U/mL penicillin, and 50 μg/mL streptomycin, and cultured in a humidified atmosphere of 5% CO2 at 37 °C (LamSystems, Myass, Russia).

### 4.3. Antibodies

Primary antibodies raised against the following proteins were used for Western blotting: Rad51, Mdm2, Cyclin A2, pCdk Tyr 15, and H2AX (Abcam, Cambridge, MA, USA), beta-actin (Gene Script, Piscataway, NJ, USA), pH2AX S139, PARP, Rad51 (Santa Cruz Biotechnology, Santa Cruz, CA, USA), pH3 S10, p53 S15, total and cleaved forms of PARP, caspase-3, Chk1, Chk2, pChk1 S 345, pChk2, and Thr68 (Cell Signaling, Danvers, MA, USA) and PARP (Invitrogen, Carlsbad, CA, USA). HRP-conjugated secondary antibodies for Western blotting were purchased from Santa Cruz Biotechnology. For immunofluorescence staining, Cy3-labeled (Jackson ImmunoResearch, West Grove, PA, USA) or Alexa Fluor-488-labeled and 647-labeled secondary antibodies (Invitrogen) were used.

### 4.4. Cellular Survival MTS-Based Assay

The normal and cancer cells indicated above were seeded into 96-well flat-bottomed plates (Corning Inc., Corning, NY, USA) and allowed to attach and grow for 24 h. The cells were then cultured for 48–72 h with the indicated concentrations of 2-APCAs, chemotherapeutic agents, or DMSO (control). Finally, the MTS reagent (Promega, Madison, WI, USA) was added to the culture medium for at least 1 h to assess the number of living cells. Cell viability was analyzed at 492 nm on a MultiScan FC plate reader (Thermo Fisher Scientific, Waltham, MA, USA). The half-inhibitory concentration (IC50) was determined using the Excel software. Data were normalized for the DMSO-treated (e.g., control) group.

### 4.5. Tubulin Polymerization Assay

The ability of 2-APCAs to interfere with the microtubules dynamics and tubulin polymerization was tested using the Tubulin Polymerization kit (Cytoskeleton Inc., Denver, CO, USA) as specified by the manufacturer. Results were obtained on a SpectraFluor Plus microplate reader (Tecan GmbH, Grödig Austria) and readings were taken every minute for 1 h (61 measurements in total).

### 4.6. Single-Cell Electrophoresis (Comet Assay)

The formation of DNA double-strand breaks (DSBs) were assessed by the alkaline version of single-cell electrophoresis (Comet Assay). The cell suspension was mixed with LMAgarose (Trevigen, Gaithersburg, MD, USA) in a ratio of 1 to 10 (30 to 300 μL, respectively) and incubated in a lysis solution overnight. The slides were further placed into an alkaline buffer solution (200 mM NaOH, 1 mM EDTA (pH > 13)) for 1 h and followed by a horizontal electrophoresis for 40 min, 25 V, and 300 mA. Next, the slides were washed twice for 1 min with deionized water and fixed in 70% ethanol for 5 min. SYBR Green I (Trevigen, Gaithersburg, MD, USA) (10,000×) was diluted with a TE buffer (10 mM Tris-HCl (pH 7.5), 1 mM EDTA). Finally, the slides were examined by fluorescent microscopy (Olympus BX63, Tokyo, Japan). The tail moment (TM) and olive tail moment (OTM)) were calculated using the ImageJ software. At least 50 comets were analyzed for each experiment. Differences between the control and treated cells in the tail moment (TM) and olive tail moment (OTM) were analyzed by the Kruskal-Wallis test followed by Dunn’s test with the Benjamini-Hochberg adjustment in R software (R Foundation for Statistical Computing, Vienna, Austria; URL https://www.R-project.org/).

### 4.7. Western Blotting 

To prepare the whole-cell extracts, the cells were lysed by a RIPA buffer (25 mM Tris-HCl pH 7.6, 150 mM NaCl, 5 mM EDTA, 1% NP-40, 1% sodium deoxycholate, 0.1% SDS), containing the cocktail of protease and phosphatase inhibitors. The cellular lysates were incubated for 20 min at 4 °C and then clarified by centrifugation for 30 min at 13,000 rpm at 4 °C. Protein concentrations were measured by the BCA assay (Thermo Fisher Scientific, Rockford, IL, USA). The samples containing 30 μg of protein were resolved on 4 to 12% Bis-Tris or 3 to 8% Tris-acetate NuPAGE gels (Invitrogen, Carlsbad, CA, USA). SDS-PAGE was carried out at 4 °C for approximately 3 h using a constant voltage (80 V) in a 1 X NuPAGE MOPS SDS running buffer (Invitrogen, Carlsbad, CA, USA). The protein transfer to a nitrocellulose membrane was performed using a 1 × transfer buffer 1 × (25 mM Tris, 192 mM Glycine, 20% (*v*/*v*) methanol, (pH 8.3)) at 350 mA for 1.5 h at 4 °C. To block the non-specific interaction, we used 5% non-fat dry milk and incubated a nitrocellulose membrane overnight with the primary antibody, and further with an HRP-conjugated secondary antibody for 1 h. Protein expression was detected by the chemiluminescence imaging system Fusion Solo S (Vilber Lourmat, Collégien, France). The densitometric analysis of Western blotting images was performed using the NIH ImageJ software (Bethesda, MD, USA).

### 4.8. Immunofluorescence Staining

Cells were seeded on glass coverslips coated with poly-L-lysine (Sigma-Aldrich, St. Louis, MO, USA) and allowed to attach for 48 h before the treatment. After washing with PBS (twice), the cells were fixed with 4% formaldehyde diluted in 1 × PBS for 15 min at room temperature. The cells were further washed three times with PBS (for 5 min each) and incubated in a blocking solution (1 × PBS, 5% normal goat serum, 0.3% Triton X-100) for 60 min at room temperature. Then, the primary rabbit antibodies Phospho-Histone H3 (Ser10) (D2C8) conjugated with secondary antibodies Alexa Fluor 488 (Cell Signaling, Danvers, MA, USA) diluted in an Antibody Dilution Buffer (1 × PBS, 1% bovine serum albumin, 0.3% Triton X-100) were added to the cells (overnight at 4 °C). The next day, the cells were washed three times with PBS for 5 min each. After a brief DAPI (Sigma-Aldrich, St. Louis, MO, USA) staining, the coverslips were mounted on glass slides and cells were visualized on a fluorescence microscope “Olympus BX63” (Olympus Corp, Tokyo, Japan). Finally, the images were captured using a Spot advanced imaging system.

### 4.9. Real-Time Monitoring of Cell Proliferation

The growth speed of RD cells cultured in the presence of PTX, Vin, or 2-APCAs were analyzed using the iCELLigence system (ACEA Biosciences, San Diego, CA, USA). For this purpose, cancer cells were seeded in electronic microtiter plates (E-Plate; Roche Diagnostics, GmbH, Mannheim, Germany) for 24 h prior to the treatment which was performed in triplicate experiments. Cells were cultured with the chemotherapeutic agents indicated above for 48–72 h. Cell index (CI) measurements were performed for every 30 min until the end time-point of the experiment. Normalized cell index (NCI) values were analyzed using the RTCA software (Roche Diagnostics, GmbH, Mannheim, Germany).

### 4.10. Molecular Docking

In order to identify the potential binding sites of 2-АРСАs on the tubulin, the molecular docking procedure was performed via the AutoDock 4.2 program package [58]. The Lamarckian genetic algorithm was used to find the tubulin-2-АРСАs supramolecular complex with minimal free energy. Tubulin was regarded as a rigid molecule; the dihedral variation was allowed for the 2-APCA molecules. The tubulin structure has been taken from the Protein Data Bank (no. 1 tub). Initial ligand structures were optimized using CAMB3LYP/def2-TZVP [59,60] by means of the PC GAMESS v. 12 program package [61]. To determine the possible interactions of 2-АРСАs with the tubulin molecule, at first, “blind docking”, without assigning a specific binding site, was carried out. “Blind docking” simulations were performed for 126 Å × 80 Å × 100 Å cell, centered on tubulin with 0.800 Å step. Each docking experiment consisted of 50 separated runs that were set to terminate after a maximum of 25 million energy evaluations. The 2-АРСАs-tubulin complexes were sorted into groups according to RMS (the root mean square difference in coordinates between this conformation and the cluster reference). To clarify the APCA binding site with 1 tub, re-docking was performed in the GOLD v.2020.1 software. All the atoms within a radius of 20 Å from the ligand were selected to specify the localization of APCA in the cavity. For all ligands, 50 separate runs were performed, as in the case of blind docking. GoldScore with a rescore by ChemScore was chosen as a function to evaluate the binding efficiency. Molecular graphics and analyses were performed with the UCSF Chimera [62].

### 4.11. Statistics

All the experiments were repeated a minimum of three times. The results are presented as the mean ± standard error (SE) for each experimental group. Differences were considered significant at *p* < 0.05.

## Figures and Tables

**Table 1:** IC50 values for 2-amino-pyrrole-carboxamides (2-APCAs) (I-IV), paclitaxel, and vinblastine for non-transformed fibroblasts (BJ tert) and soft tissue sarcoma cell lines.

Sarcoma Cell Lines	2-APCA-I (µmol)	2-APCA-II (µmol)	2-APCA-III (µmol)	2-APCA-IV (µmol)	Paclitaxel	Vinblastine
BJ tert	11.3 ± 2	38.3 ± 3.3	6.2 ± 0.5	54.3 ± 2.8	57.2 ± 3.7 nmol	45.3 ± 5.9 nmol
HT1080	8.2 ± 0.2	30.6 ± 3.5	13.8 ± 2.6	60 ± 5.5	57.2 ± 6.7 pmol	18.9 ± 2 pmol
RD	4.2 ± 0.5	5.2 ± 0.4	2.5 ± 0.2	7.9 ± 0.7	9.8 ± 0.7 nmol	1.1 ± 0.1 nmol
SK-LMS-1	7.1 ± 0.2	12.5 ± 0.6	11.3 ± 0.9	15.4 ± 2.4	23.9 ± 3.3 nmol	20 ± 2.4 nmol
А673	28.9 ± 1.3	27.1 ± 3.8	10.2 ± 1.1	64.3 ± 6.4	18.8 ± 4.4 pmol	740.4 ± 174.9 fmol
U2OS	12 ± 0.13	28.8 ± 1.1	10.6 ± 0.7	63.1 ± 8.5	15.8 ± 2.8 nmol	124.2 ± 22.9 nmol

**Table 2:** IC50 values for 2-APCAs (I-IV), paclitaxel, and vinblastine for epithelial cancer cell lines.

Epithelial Cancer Cell Lines	2-APCA-I (µmol)	2-APCA-II (µmol)	2-APCA-III (µmol)	2-APCA-IV (µmol)	Paclitaxel	Vinblastine
HCC1806	13.5 ± 2.3	23.7 ± 1.1	12.4 ± 1	29.2 ± 5.7	31.6 ± 6 pmol	322.3 ± 36.5 pmol
MDA-MB-231	20.3 ± 4	29.5 ± 4.8	7.2 ± 0.3	19.7 ± 1.3	35 ± 10.9 pmol	852.6 ± 271 pmol
Н1299	29.9 ± 5.7	29.5 ± 4.8	5 ± 0.1	5 ± 0.1	1.7 ± 0.5 pmol	6.7 ± 1.9 pmol
РС-3	15.8 ± 3.5	43.1 ± 0.9	10.7 ± 1.1	32.2 ± 4.5	42 ± 9.6 pmol	40.4 ± 10.8 fmol
Hela	2.8 ± 0.4	33 ± 2	14.4 ± 2	33.9 ± 3.2	352.4 ± 51 pmol	1.7 ± 0.4 pmol

**Table 3:** IC50 values for 2-APCAs (I-IV), paclitaxel, and vinblastine for cancer cells lines sensitive vs. resistant to imatinib mesylate (IM-R), or paclitaxel (Tx-R).

Cell Lines	2-APCA-I (µmol)	2-APCA-II (µmol)	2-APCA-III (µmol)	2-APCA-IV (µmol)	Paclitaxel	Vinblastine
**GIST T1**	1.3 ± 0.2	8.3 ± 0.3	2.6 ± 0.1	8.4 ± 0.5	51.7 ± 9.5 pmol	109.1 ± 18.9 pmol
**GIST T1-IM-R**	9.3 ± 0.4	9.7 ± 0.3	2 ± 0.1	13.8 ± 0.9	7.8 ± 1 pmol	481.3 ± 77.4 pmol
**GIST T1-Tx-R**	6.7 ± 0.2	9.9 ± 0.2	4.6 ± 0.3	12.4 ± 1.2	431.6 ± 69.8 nmol	41.8 ± 0.6 nmol
**GIST 430**	8.9 ± 0.5	9 ± 0.5	8.9 ± 0.3	9 ± 0.5	30.4 ± 7.6 µmol	33.4 ± 6.5 µmol
**HCC1806**	13.5 ± 2.3	23.7 ± 1.1	12.4 ± 1	29.2± 5.7	31.6 ± 6 pmol	322.3 ± 36.5 pmol
**HCC1806 Tx-R**	21.8 ± 1.9	13.5 ± 0.7	10.2 ± 1.2	22.3 ± 2.1	949 ± 78.8 nmol	415.8 ± 81.7 nmol

**Table 4:** The amino acid composition of the 2-APCAs binding sites in tubulin.

2-APCA	Amino Acid Composition of the Binding Site
**2-APCA-I**	A: ARG-402, ALA-403, HIS-406 B: PHE-262, PRO-263, ARG-264, HIS-266, TRP-346, GLU-431, TYR-432, GLN-434, TYR-435, GLN-436
**2-APCA-II**	LEU-26, GLU-27, HIS-28, ASP-33, GLY-34, GLN-35, MET-36, SER-236, ALA-240, SER-241, PHE-244, ARG-320, ASN-356, TYR-357, GLU-358, PRO-359, THR-361
**2-APCA-III**	ALA-19, TRP-21, GLU-22, LEU-23, CYS-25, LYS-40, THR-41, ILE-42, ARG-84, GLN-85, LEU-86, PHE-87, ARG-229, GLN-233, VAL-362, VAL-363, PRO-364, ASP-367
**2-APCA-IV**	PRO-274, THR-276, SER-280, GLN-281, GLN-282, TYR-283, ARG-284, ALA-285, LEU-286, GLU-290, GLY-370, LEU-371, LYS-372

**Table 5:** Results of the docking studies with GOLD fitness values (gold fitness) and predicted binding free energies (Chemscore.dG).

	Gold Fitness	ChemScore dG (kJ·mol^−1^)
2-АРСА-I	53.39	−26.32
2-АРСА-II	52.54	−26.37
2-АРСА-III	65.43	−27.71
2-АРСА-IV	57.91	−25.00

## Data Availability

The data presented in this study are included in this published article.

## Supplementary Materials

The following are available online. Figure S1: Characteristics of the resistant cancer cells; Figure S2: 2-APCA-III induces dysregulcation in cell cycle of cancer cell lines; Figure S3: 2-APCA induce apoptosis of cancer cell lines in a dose-dependent manner.

## Sample Availability

Samples of the compounds are not available from the authors.
