# Supplementary material for: 2-APCAs, the Novel Microtubule Targeting Agents Active against Distinct Cancer Cell Lines
Source: Molecules. 2021 Jan 25;26(3):616. doi: 10.3390/molecules26030616 (PMC7865999; doi:10.3390/molecules26030616)

Supplementary Figure S1. Characteristics of the resistant cancer cells. (A-B) MTS-based viability assay in PTX-treated HCC1806 vs. HCC1806-Tx-R cells and PTX-treated GIST-T1 vs. GIST T1-Tx-R (R, resistant) cells. Cells were treated with the indicated concentrations of PTX and assessed 48 h of post-treatment. Data was normalized to DMSO-treated controls; (C) Expression of the multidrug resistance proteins (MDR-1 and MRP-1) in the naïve- and resistant HCC1806 breast cancer and GIST cell lines. (D-E) Growth-inhibitory curves of sensitive and resistant to Paclitaxel (Tx-R) HCC1806 breast cancer (D) and T-1 gastrointestinal stromal tumor (E) cell lines.

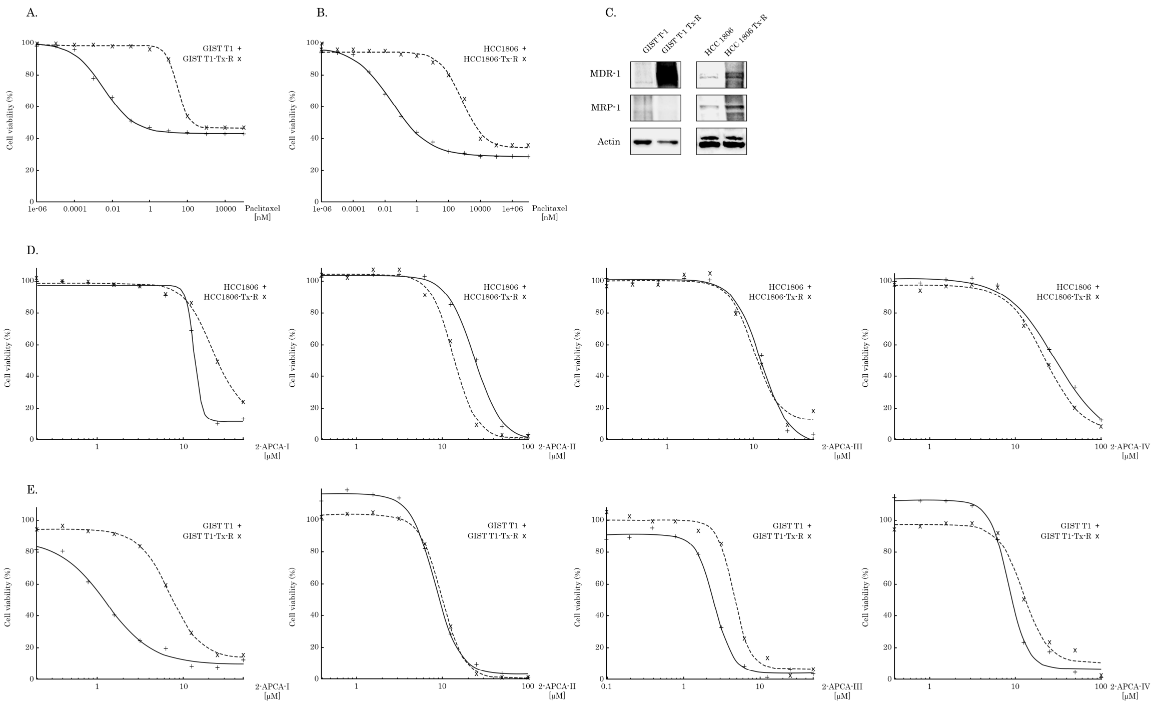

Supplementary Figure S2. Expression of cyclin A2, MDM-2, phospho-Cdk2 (Tyr15), and phospho-H3 (Ser10) in H1299 (A) and MDA-MB-231 (B) cancer cells treated with 2-APCA-III or Paclitaxel (PTX) for 48 h. Actin stain was used for loading control.

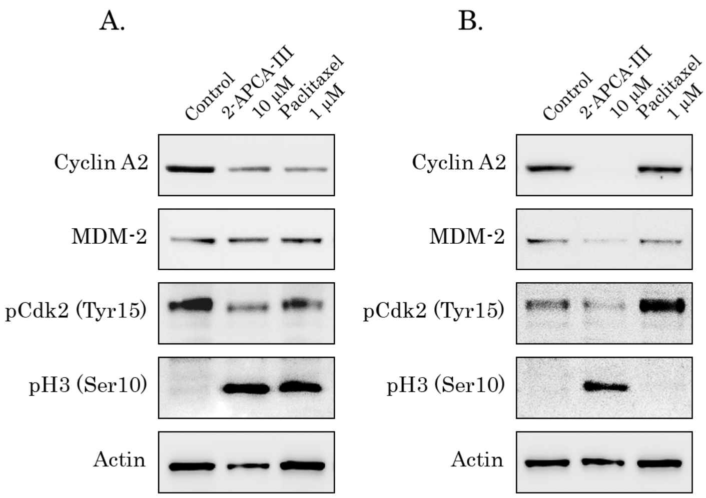

Supplementary Figure S3. 2-APCA induce apoptosis of cancer cell lines in a dose-dependent manner. Left panel - Immunoblot analysis for apoptosis markers (cleaved forms of PARP and caspase-3), Rad51 recombinase and RD rhabdomyosarcoma (A), SK-LMS-1 leiomyosarcoma (C), and GIST T-1R cells (E) after treatment with DMSO (negative control), 2-APCA I-IV and PTX for 72 h. Actin stain used as a loading control. Right panel - Densitomentric analysis of protein expression shown on western blots for RD (B), SKL-MS-1 (D) and GIST T-1R (F) cells.

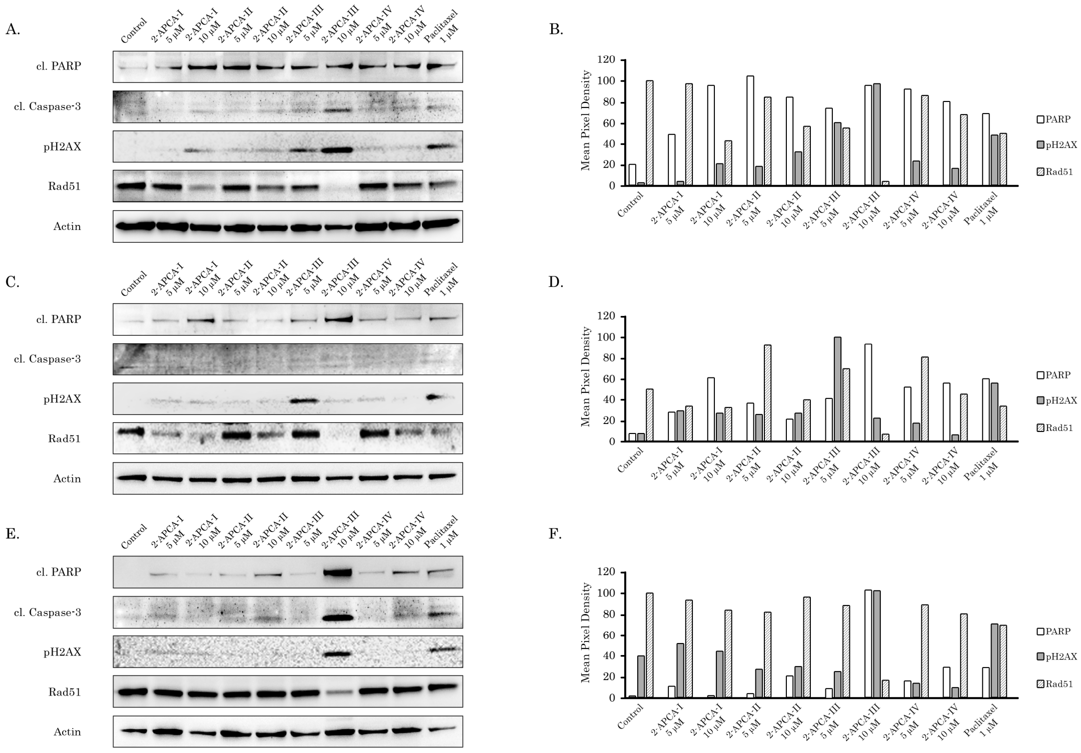

Supplement: Supplementary file 1 [file molecules-26-00616-s001.pdf]
